# A Robust Gene Expression Prognostic Signature for Overall Survival in High-Grade Serous Ovarian Cancer

**DOI:** 10.1155/2019/3614207

**Published:** 2019-11-07

**Authors:** Yue Zhao, Shao-Min Yang, Yu-Lan Jin, Guang-Wu Xiong, Pin Wang, Antoine M. Snijders, Jian-Hua Mao, Xiao-Wei Zhang, Bo Hang

**Affiliations:** ^1^Department of Gynecology, The First Affiliated Hospital, Nanjing Medical University, Nanjing 210000, China; ^2^Department of Obstetrics and Gynecology, Peking University Third Hospital, Beijing 100191, China; ^3^Department of Pathology, School of Basic Medical Sciences, Third Hospital, Peking University Health Science Center, Beijing 100191, China; ^4^Department of Pathology, Beijing Obstetrics and Gynecology Hospital, Capital Medical University, Beijing Maternal and Child Health Care Hospital, Beijing 100026, China; ^5^Women & Children Health Center, The Third Affiliated Hospital of Chongqing Medical University, Chongqing 401120, China; ^6^Department of Gastroenterology, Nanjing Drum Tower Hospital, Nanjing University Medical School, Nanjing, Jiangsu 210008, China; ^7^Biological Systems and Engineering Division, Lawrence Berkeley National Laboratory, Berkeley, CA 94720, USA

## Abstract

The objective of this research was to develop a robust gene expression-based prognostic signature and scoring system for predicting overall survival (OS) of patients with high-grade serous ovarian cancer (HGSOC). Transcriptomic data of HGSOC patients were obtained from six independent studies in the NCBI GEO database. Genes significantly deregulated and associated with OS in HGSOCs were selected using GEO2R and Kaplan–Meier analysis with log-rank testing, respectively. Enrichment analysis for biological processes and pathways was performed using Gene Ontology analysis. A resampling/cross-validation method with Cox regression analysis was used to identify a novel gene expression-based signature associated with OS, and a prognostic scoring system was developed and further validated in nine independent HGSOC datasets. We first identified 488 significantly deregulated genes in HGSOC patients, of which 232 were found to be significantly associated with their OS. These genes were significantly enriched for cell cycle division, epithelial cell differentiation, p53 signaling pathway, vasculature development, and other processes. A novel 11-gene prognostic signature was identified and a prognostic scoring system was developed, which robustly predicted OS in HGSOC patients in 100 sampling test sets. The scoring system was further validated successfully in nine additional HGSOC public datasets. In conclusion, our integrative bioinformatics study combining transcriptomic and clinical data established an 11-gene prognostic signature for robust and reproducible prediction of OS in HGSOC patients. This signature could be of clinical value for guiding therapeutic selection and individualized treatment.

## 1. Introduction

Ovarian cancer (OC) represents the most lethal gynaecological malignancy and the fifth leading cause of death in women, with a 5-year survival rate around 10% [1]. Due to lack of early screening and diagnostic measures, most patients are diagnosed with OC at an advanced stage. Globally, more than 239,000 women are diagnosed with OC and 152,000 succumb to this disease each year [2].

OC has been shown to have considerable complexity and heterogeneity in biology, drug response, and survival time [3], representing a major obstacle for its precision medicine practice. OCs of epithelial origin constitute approximately 90% of all the cases, whereas ovarian sex cord stromal tumor, ovarian germ cell tumor, and secondary tumor of ovarian metastasis (e.g., Krukenberg tumor) are less frequent [4]. High-grade serous ovarian carcinoma (HGSOC) is the most predominant in epithelial OCs, accounting for 70–80% of OC deaths [5]. The majority of HGSOCs can be grouped into four subtypes based on gene overexpression levels specific for each subtype: mesenchymal, immunoreactive, differentiated, and proliferative [6].

HGSOC has been characterized by both genetic alterations, including inherited BRCA gene mutations, TP53 mutations, DNA damage, chromosomal instability [6, 7], and changes in RNA and miRNA expression and methylation status [8]. Microarray and next-generation sequencing technologies have become vital tools for identifying these changes genomewide, providing novel opportunities for the identification of biomarkers for diagnosis, prognosis, therapeutic targets, and treatment response. For instance, many multigene biomarkers based on transcription patterns have been associated with prognosis across tumor types [9–14]. A number of groups have sought to use genomewide gene expression data to identify multigene signatures aimed at predicting clinical outcomes, therapy responses, and subtypes in OC [13–18]. Many existing signatures were generated using partial genome annotations, limited number of patients, or used targeted gene selecting. Thus, it is very much warranted to identify and develop clinically valuable gene signatures for OC prognosis, especially when based on comprehensive and unbiased whole-genome data.

In this study, we employed a multistep bioinformatic strategy that uses omics information and clinical data to build a gene expression prognostic scoring system in HGSOC. We previously developed this approach to identify and successfully validate a 53-gene signature associated with OS of gastric cancer [11] and a 27-gene signature for lung adenocarcinoma [12]. Here, we used fifteen publicly available datasets of HGSOCs; six were used to identify an 11-gene signature associated with patient prognosis using Cox regression analysis and cross-validation. We then used nine independent HGSOC datasets to validate the prognostic scoring system and signature's performance. Moreover, in comparison with an existing 5-gene expression signature for ovarian serous cystadenocarcinoma (CAC) [15], we showed that our signature was superior in determining overall survival for this type of epithelial ovarian carcinoma.

## 2. Materials and Methods

### 2.1. Patient Datasets

To broadly mine all the available information on HGSOCs, we have screened and used 15 independent datasets in the current study. Six public datasets from the NCBI Gene Expression Omnibus (GSE18520, GSE26712, GSE40595, GSE38666, GSE27651, and GSE2328) provided the HGSOC gene transcript data to identify genes differentially expressed between tumor and normal ovarian tissues. TCGA HGSOC data were used to identify the gene signature and develop the prognostic scoring system for predicting OS of patients. Nine additional datasets (GSE32063, GSE19829 GPL570, GSE30161, GSE3149, OV-AU-ICGC, GSE14764, GSE9891, GSE 17260, and GSE32062) were used for independent validation of the gene signature and prognostic scoring system.

### 2.2. Statistical Analysis

By employing a 1.5-fold change cutoff and adjusted *p*-value <0.05, the differentially expressed genes between normal versus HGSOC tissues were identified with GEO2R. Differentially expressed genes associated with OS in patients with HGSOC were selected using KM survival analysis (Kaplan–Meier plotter (http://kmplot.com)) with a hazard ratio (HR) with 95% confidence intervals and log-rank *p* value cutoff for each gene at 0.05 [19].

### 2.3. Gene Ontology Pathway Analysis and Network Construction

Metascape (http://www.metascape.org) was used to assess overrepresentation of Gene Ontology categories in biological networks [20]. Cytoscape 3.4.0 (http://www.cytoscape.org) was applied to generate and visualize the gene coexpression networks, to better understand the biological processes enriched, as well as their relationships in the form of a network instead of the tabular text format [21, 22]. Note that KEGG pathway (http://www.genome.jp/kegg), GO Biological Processes (http://geneontology.org), Reactome Gene Sets (http://www.reactome.org), and CORUM (http://mips.helmholtz-muenchen.de/corum) were ontology sources of gene network, pathway, and process enrichment analysis.

### 2.4. Gene Expression Signature-Based Prognostic Risk Score

Clinical data of HGSOC patients were obtained from the TCGA dataset (http://cancergenome.nih.gov), with which a biomarker panel associated with OS was reachable. 100 random selections of 307 patients from TCGA were conducted and used as training sets. The remaining patients for each selection were used as test sets to validate the reliability of the identified biomarker panel for prognosis.

Forward conditional Cox regressions using SPSS were carried out on each of the 100 training sets in order to isolate the biomarker panel. Selected genes were recorded and those that appeared in at least 20% of 100 training sets were included in our biomarker panel. Subsequently, Cox regression was repeated on all 100 training sets using our 11-gene signature as covariates and using the forced entry (enter) method to obtain the coefficient values for each biomarker. 100 coefficients for every gene in the biomarker panel were then obtained, and the average of them was used to estimate the true coefficient of each gene. A formula was created to act as the prognostic scoring system, and all the patients can get their scores accordingly:

(1) $$\begin{array}{c} \overset{11}{\underset{i=1}{\sum }}\left(\text{gene }i\text{ coefficient}\right)\;*\;\left(\text{gene }i\text{ expression level}\right). \end{array}$$

The patients in the training sets were ranked by their prognostic scores and divided into three equal-sized cohorts. The corresponding prognostic scores at cut points were recorded and averaged as the true cut point scores, with which the patients in the test sets were also split into three groups: “good”, “intermediate”, and “poor” groups. Differences in OS among the three groups in all the test sets were determined by constructing Kaplan–Meier plots and performing log-rank tests.

### 2.5. Validation in Independent Datasets and Comparison with an Existing Signature

The 11-gene biomarker panel was further validated in nine independent datasets ([Supplementary-material supplementary-material-1]). New coefficients for the 11 genes were obtained from Cox regression. Prognostic scores for all patients were calculated, and patients were ranked based on their scores and divided into three equal-sized cohorts. Kaplan–Meier analysis and a log-rank test were conducted to determine differences in survival, as previously described [11, 12].

We compared the performance of our 11-gene signature with a recently published 5-gene signature for prognosis of ovarian serous CAC [15]. A multivariate Cox regression analysis was conducted with the 5 genes on the same 100 training sets as described above for our inner validation. Coefficients for each of the 5 genes used in [15] and scores of all 307 patients were calculated as above. Then patients were divided into tertiles (good, intermediate, and poor) based on their prognostic scores, and the cut point scores were recorded and averaged. Kaplan–Meier analysis was performed, and a log-rank test was used to demonstrate differences in OS among different groups for all test sets.

## 3. Results

### 3.1. Identification of Deregulated Genes in HGSOCs

**Figure 1:**
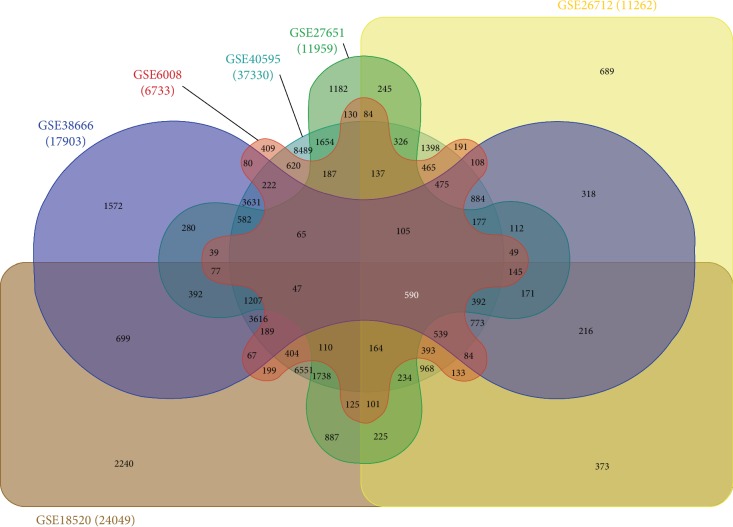
Human datasets of ovarian cancer and normal sample tissues. Samples were obtained from six independent gene transcript datasets containing HGSOC and normal ovarian cases. To identify genes (common probe IDs) consistently deregulated in HGSOC, a fold-change cutoff of 1.5 and adjusted *p*-value <0.05 were used for each dataset.

To identify genes that are consistently deregulated in HGSOC, we performed a meta-analysis and compared gene transcript levels in six publically available datasets containing transcriptomic data for both HGSOC and normal ovarian tissues (*n* = 397 from GSE18520, GSE26712, GSE40595, GSE38666, GSE27651, and GSE2328) using GEO2R. For each dataset, we compared HGSOC gene expression to gene expression in normal ovarian tissues (Figure 1).

The criteria for significant differential expression for each gene were set to a 1.5-fold change and adjusted *p*-value <0.05. A total of 562 probe IDs (260 downregulated and 302 upregulated) were consistently up- or downregulated across all six datasets, representing 488 unique genes (222 downregulated and 266 upregulated) (Figure 1 and [Supplementary-material supplementary-material-1]).

### 3.2. Analysis of Deregulated Genes and Overall Survival of HGSOCs

**Figure 2:**
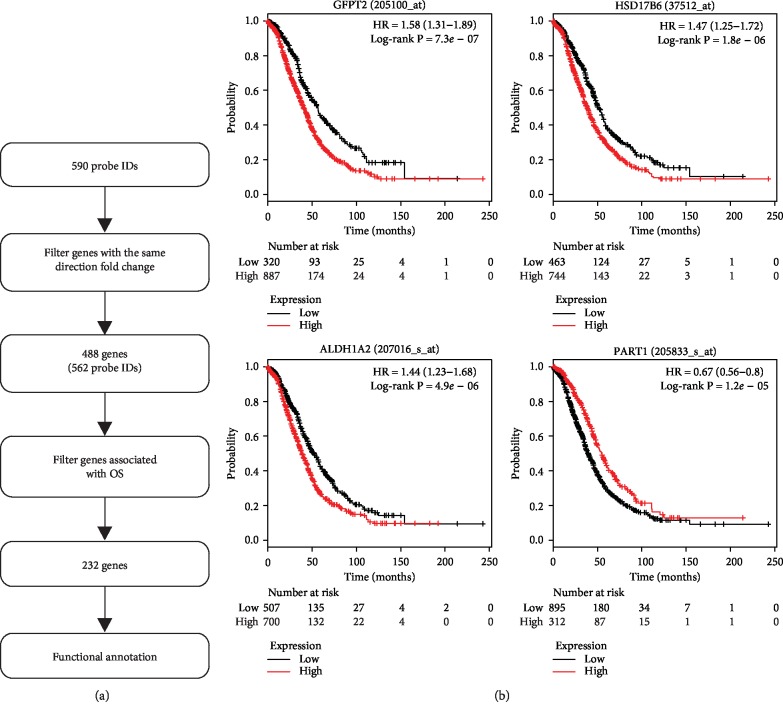
Identification of genes associated with prognostic function in HGSOC. (a) The 562 consistently deregulated probe IDs identified represent 488 genes in the cancer patients. Through Kaplan–Meier survival analysis, 232 genes were found to be significantly associated with overall survival of HGSOC patients. Functional annotation was carried out for the 232 genes. (b) Examples of Kaplan–Meier survival curves for four individual genes significantly associated with overall survival in HGSOC patients, which was divided into two groups to maximize the difference in survival using log-rank testing between groups. We used HR and log-rank *p*-value for the curve comparison between the groups.

The prognostic value for each of the 488 deregulated genes individually in HGSOC patients was evaluated in a large public clinical database which integrates gene expression and patient survival using Kaplan–Meier survival analysis (Figure 2(a)). The effects of high or low expression levels of these genes on OS were assessed using Kaplan–Meier survival analysis and compared statistically using the log-rank test, with representative genes shown in Figure 2(b). The results showed that 232 out of the 488 genes were significantly associated with OS (adjusted *p*-value <0.05; Figure 2(b) and [Supplementary-material supplementary-material-1]). The hazard ratio (HR) for 82 genes was <1 (higher gene expression associated with good prognosis), which are referred as protective genes, whereas 150 genes had a HR >1 (higher gene expression associated with poor prognosis), which are considered risk genes.

### 3.3. Gene Ontology (GO) Analysis of Prognostic Genes in HGSOC

**Figure 3:**
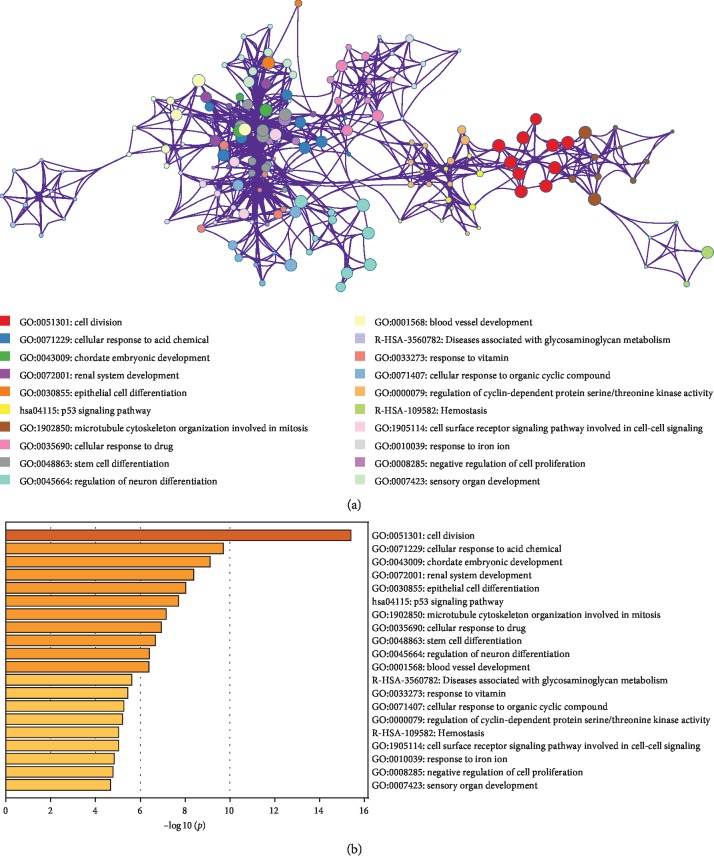
Gene Ontology analysis of 232 genes associated with OS. (a) Network layout of the clusters generated with the complete list of the 232 OS-associated genes in HGSOC. Each node represents one enriched term, where its size is proportional to the number of genes associated with each term, and its color representing its cluster identity (i.e., nodes of the same color belong to the same cluster). All similar terms with a kappa similarity score >0.3 are connected by edges (the thicker the edge, the higher the similarity). One term from each cluster was selected to describe the general function of each cluster. Created by Metascape (http://metascape.org). (b) Top 20 most significant GO categories associated with the 232 genes.

To understand the potential biological functions of the 232 genes significantly associated with OS in HGSOC patients, we conducted Gene Ontology (GO) analysis using Metascape and found significant enrichment of many cellular process and pathway-related genes associated with cancer development including cell division, epithelial cell differentiation, p53 signaling pathway, and vasculature development (Figure 3(a) and [Supplementary-material supplementary-material-1]). The interconnectivity of related GO terms was visualized using Cytoscape where individual GO terms are displayed as nodes connected based on similarity (Figure 3(b)).

### 3.4. Establishment of an 11-Gene Prognostic Scoring System in HGSOCs

**Figure 4:**
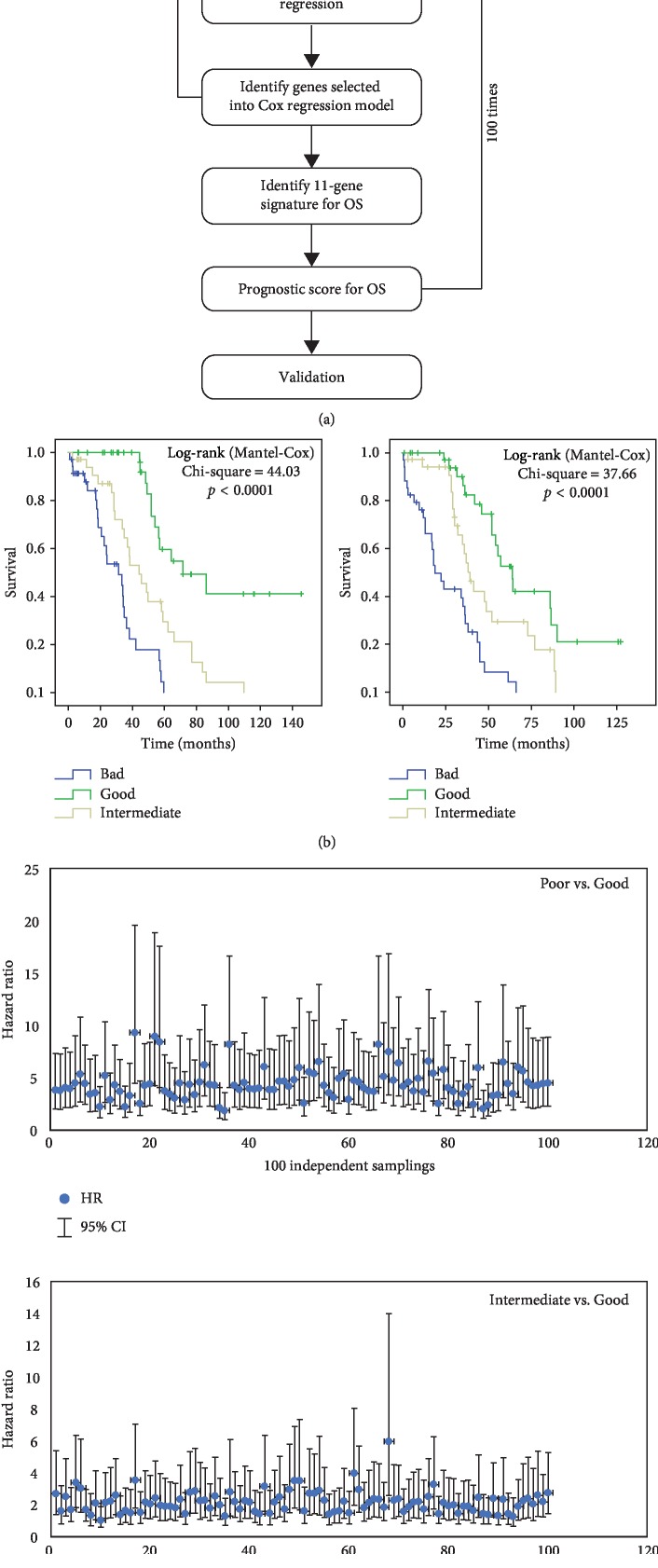
Strategy to generate an 11-gene prognostic signature and its performance evaluation. (a) We employed multivariate Cox regression analysis on 100 training sets through random sampling for the 232 genes and identified 11 genes selected into our Cox regression model. Such a signature was used to generate a prognostic scoring system, which was further validated using 100 randomly assembled test sets. (b) Representative Kaplan–Meier overall survival curves in two test sets. These curves were separated into tertiles according to the prognostic score calculated using the 11-gene signature. (c) HR values and their 95% confidence interval across the 100 test sets, calculated using a Cox model based on the prognostic score comparing poor vs. good (top) and intermediate vs. good (bottom).

Figure 4(a) shows the strategy we employed to isolate a prognostic biomarker signature and to develop a scoring system based on the 232 genes that were found to be significantly associated with OS in HGSOC patients. We first used a random resampling method to split 307 patients from the TCGA dataset into 100 training (200 patients) and 100 testing (107 patients) sets. The training sets were then used to isolate a prognostic signature, and the testing sets were used for validation. First, we performed a multivariate Cox regression analysis in all 100 training sets to identify statistically significant independent genes within the 232 genes for predicting OS. Genes that recurred in at least 20% of 100 training were assembled into an 11-gene signature: *RAD51AP1*, *CADPS2*, *DSE*, *ITGB8*, *PDE10A*, *GALNT10*, *SNX1*, *MTHFD*2, *C9orf16*, *PYCR1*, and *ARL4* ([Supplementary-material supplementary-material-1]). For each of the 11 genes in the signature, gene function and known roles in ovarian and other cancers are summarized in [Supplementary-material supplementary-material-1].

A prognostic score for each cancer patient was used to assess each patient's risk of death and was defined as the linear combination of logarithmically transformed gene expression levels weighted by average Cox regression coefficients ([Supplementary-material supplementary-material-1]) obtained from 100 training datasets [11, 12]. The prognostic scores were assigned for all patients in both training and test sets. In each training set, the patients were divided into tertiles based on their prognostic score. The cutpoint scores were recorded and averaged for each of 100 training sets. Based on the average scores, each test set was split into three groups, i.e., good, intermediate, and poor. We then performed Kaplan–Meier and log-rank test analysis to determine significant differences in OS among different groups for all test sets (Figure 4(b)). The hazard ratios (HR) for the “intermediate” and “poor” groups in comparison with the “good” groups were calculated for each test set. In 99% of the test sets, patients in the “poor” groups had a significantly shorter OS than those in the “good” groups (HR confidence interval above “1”) (Figure 4(c), top panel), while in more than 60% of the test sets, patients in the “intermediate” groups showed a significantly shorter OS than those in the “good” groups (Figure 4(c), bottom panel). These results validated the discriminative ability of this 11-gene signature and prognostic scoring system to stratify patients with good or worse prognosis.

### 3.5. Independent Validation of the 11-Gene Scoring System

**Figure 5:**
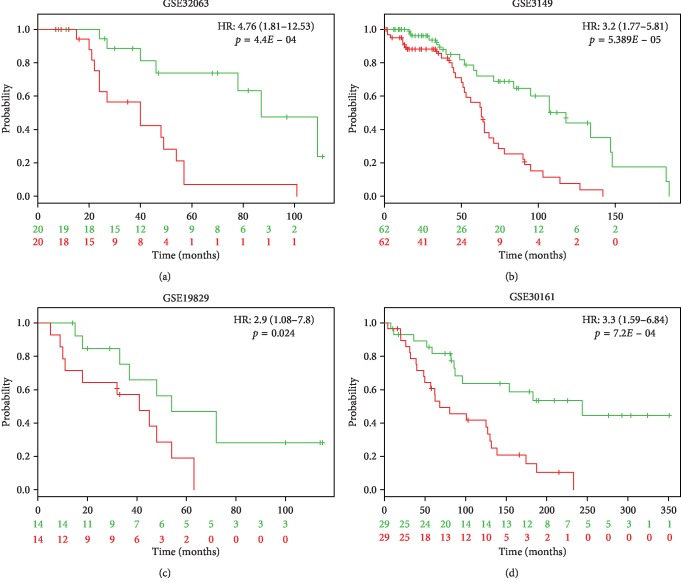
Validation of the 11-gene signature using four independent ovarian cancer cohorts. We analyzed the Kaplan–Meier plots generated for the four cohorts used by applying the 11-gene signature. The patient cohort was split by the median based on the prognostic index, and the log-rank *p*-values of the curve comparison between the risk groups and HR are shown. The HR values and 95% confidence intervals were calculated using Cox survival analysis.

To further validate our 11-gene signature, we tested it in nine independent OC datasets ([Supplementary-material supplementary-material-1]). Prognostic scores for all patients were calculated and patients were ranked based on their score. Significant differences were identified using Kaplan–Meier analysis across all nine datasets between patient cohorts of “good” and “poor” prognosis. Patients with a high prognostic score had a significantly shorter OS than those patients who scored low (*p* < 0.05) (Figure 5). The HR values range from 1.94 to 9.76 ([Supplementary-material supplementary-material-1]) We conclude that the 11-gene prognostic scoring system reproducibly predicts overall survival of HGSOC patients.

### 3.6. Comparison with an Existing Prognostic Signature

We compared the performance of our 11-gene signature with a recently published 5-gene expression signature predicting clinical outcome of ovarian serous CAC [15] by performing a multivariate Cox regression analysis using the same strategy described in Figure 4 where for 100 training sets, coefficients for each of the 5 genes and scores of all the 307 patients were calculated.

**Figure 6:**
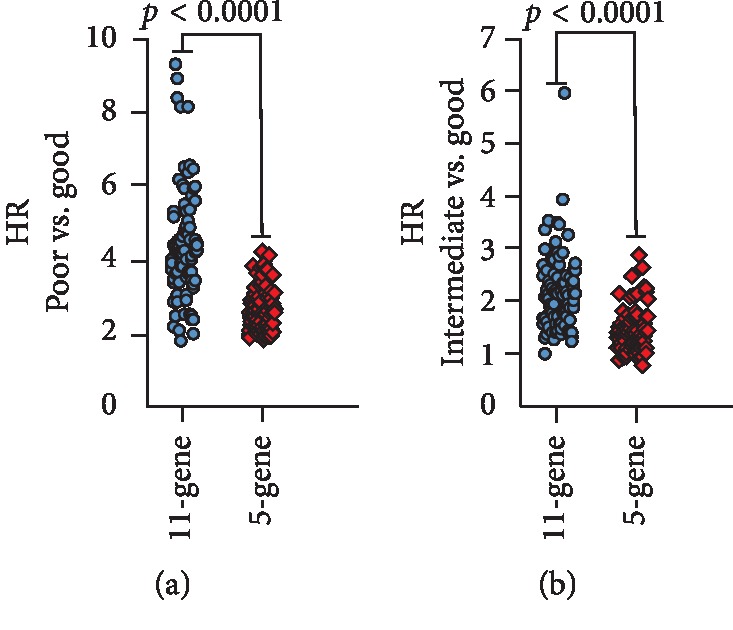
Comparison of HR and 95% confidence interval between the 11-gene and 5-gene signatures. For both 11-gene and 5-gene signatures, the HR of all the 100 test sets was calculated using a Cox model based on the prognostic score between groups (poor vs. good: top; intermediate vs. good: bottom). The differences between the two signatures were significant for both the poor vs. good groups and the intermediate vs. good groups (*p* < 0.0001).

Figure 6 shows the HR and 95% confidence interval for the “intermediate” and “poor” groups in comparison with the “good” groups in the 100 test sets. For the 5-gene panel, in 90% of the testing sets, patients in the “poor” groups had a significantly shorter OS than those in the “good” groups. For the “intermediate” groups vs. “good” groups, only in 12% of the testing sets, patients showed a significantly shorter OS. In comparison, for our 11-gene signature, these two numbers are 99% and 61%, respectively. In addition, the median HR of the 11-gene signature was on average 1.46-fold higher in the “intermediate” vs. “good” groups and 1.73-fold higher in the “poor” vs. “good” groups compared to the 5-gene signature (Figure 6). These results indicate that the 11-gene signature has discriminative ability for determining OS in ovarian CAC patients, which is also significantly superior to the 5-gene panel.

## 4. Discussion

Identification and development of reliable predictive biomarkers and new therapeutic targets are critical for significantly improving cancer patient outcomes. The objective of this work was to use a multistep bioinformatics analytic strategy we developed previously [11, 12] to analyze six publicly available omics and clinical datasets to generate a robust prognostic signature for patients with HGSOC. We first identified 232 genes associated with OS that served as candidate markers to provide a prediction of the prognosis of HGSOC patients. Eventually, we selected an 11-gene prognostic signature and scoring system showing strong discriminative power to separate patients with good or poor survival. Moreover, the results were independently validated in each of the nine independent HGSOC datasets. We also demonstrated that our 11-gene signature has higher predictive power compared to an existing prognostic panel developed for ovarian serous CAC. Taken together, the 11-gene signature could be of translational value for clinical use. We are currently working on the development of a multiplex high-throughput assay to facilitate the clinical use of the signature. To date, there are still no clinically useful prognostic biomarkers/scores in OC. However, two multigene expression-based scores, the Oncotype DX 21-gene breast cancer assay developed by Genomic Health [9, 23] and the MammaPrint 70-gene breast cancer recurrence assay by Agendia [24], have been utilized to guide treatment decisions, such as for adjuvant chemotherapy in breast cancer [23]. These two tests represent the first prognostic gene expression assays that have successfully passed multiple independent clinical trials.

Microarray and next-generation sequencing technologies broadened the accessibility of large cancer genomewide expression profiles. Taking advantage of these unbiased genomewide approaches, we established multigene signatures for predictive and prognostic purposes, including the 11-gene signature described in this study. To discover a novel panel of prognostic biomarkers is the first step in developing a practically valuable assay/score in a clinical setting. The next steps include multicenter clinical trials and prospective trials that allow further validation of the efficacy and accuracy of the signature, in order to make a successful clinical translation. It should be mentioned that microarray data-based analyses have generated many single and multiple gene biomarkers/signatures associated with prognosis of specific types of cancers including OC. For OC, several prognostic signatures have been developed based on different platforms, as described before. While these signatures can predict OC survival, some of them were developed based on limited patient numbers or conducted within a single medical center. In addition, signatures developed in earlier years were either based on incomplete genome annotations or based solely on existing knowledge. Nevertheless, we expect that with ongoing and future prospective studies, some of these preclinical biomarker signatures, including the 11-gene signature described here, will be fully evaluated for their value in the clinical settings.

Ovarian cancer, like many other cancers, occurs through the accumulation of genetic alterations, which can result in deregulation of gene expression. So far, there is still limited information on the genes that are associated with prognosis of OC. [Supplementary-material supplementary-material-1] summarizes the known functions for each gene in the 11-gene panel in tumor development and prognostic relevance. Of them, six have already been implicated in the development and progression of HGSOCs in previously published studies [25–31]. Six genes (*RAD51AP1*, *DSE*, *ITGB8*, *GALNT10*, *SNX1*, and *MTHFD2*) were reported to provide useful prognostic information about the survival in various types of cancer [25, 27, 28, 32–36], including three genes (*RAD51AP1*, *ITGB8*, and *GALNT10*) which were reported to be prognostic for OC [25, 28, 32]. *RAD51AP1*, which encodes an RAD51 accessory protein, participates in the homologous recombination DNA damage response pathway. The finding in this study is in agreement with DNA repair defects in HGSOCs. Upregulation of *RAD51AP1* predicted poorer OS in patients with ovarian cancer [25]. *DSE* (*SART2*) gene has been shown to be frequently upregulated in human brain tumors and other types of cancer [37]. Moreover, elevated *DSE* expression in glioma is associated with a worse tumor grade and poor OS [37]. Elevated levels were also detected in cervical, ovarian, and endometrial cancers [34]. *ITGB8* encodes a *β*-subunit of integrin, and integrins play a regulatory role on cancer cells through survival- and metastasis-related signaling pathways [34]. Upregulation of *ITGB8* has been shown in several types of cancers, including HGSOCs. In addition, it was found that its expression is an independent biomarker for predicting unfavorable survival of patients with HGSOCs [27]. Integrative network analysis of TCGA data has shown that *GALNT10* was highly predictive for the OS of ovarian cancer patients [28]. There is evidence that *SNX1* may play a role in tumorigenesis and its downregulation is significantly correlated with poor OS of colon cancer patients [29]. *MTHFD2* is a gene associated with cancer development, and its high expression is associated with poor prognosis of many types of cancer, for example [36–38]. Five of these genes, *CADPS2*, *MTHFD2*, *PDE10A*, *PYCR1*, and *ARL4*, have never been reported to have a role in OC ([Supplementary-material supplementary-material-1]). Interestingly, the genes in the multigene panels reported in the literature, including our 11-gene signature, are rarely overlapping, which may reflect the disparity in tumor samples, microarray designs, database selection, and analytical approaches. The genes in this signature may be novel potential therapeutic targets for HGSOCs.

The genes included in our signature might also be potential biomarkers or targets for the treatment of OC. Personalized treatment is often highlighted in today's clinical practice, where the molecular features such as genetic background of an individual patient's tumor determine the prime treatment modalities. For example, Prexasertib (LY2606368), a cell cycle checkpoint kinase 1 and 2 inhibitor, showed clinical activity and was tolerable in HGSOC patients with BRCA wild-type disease [39].

In conclusion, as the most lethal gynaecological malignancy, OC is undoubtedly a challenge for patients, medical practitioners, and researchers. In this study, with an unbiased multistep bioinformatics analytic strategy, we identified an 11-gene prognostic biomarker panel which robustly and accurately predicts overall survival in patients with HGSOCs. Gene Ontology analysis revealed several important enriched cellular processes and pathways in HGSOCs. Together, our results pave the way for developing a clinical assay for guiding therapeutic selection and individualized treatment.

## Data Availability

The data used to support the findings of this study are available from the corresponding author upon request.

## Abbreviations

OC: Ovarian cancer
HGSOC: High-grade serous ovarian cancer
CAC: Cystadenocarcinoma
HR: Hazard ratio
K-M plot: Kaplan–Meier plot
OS: Overall survival
TCGA: The Cancer Genome Atlas.

## Supplementary Materials

Supplementary Materials Table S1: summary of independent validation of the 11-gene signature in 9 datasets. Table S2: list of genes that are consistently deregulated in HGSOC across six datasets using criteria: adjusted *p* < 0.05 and fold change >1.5. Table S3: the impact of deregulated genes on overall survival (OS). Genes significantly associated with OS are highlighted in yellow. Table S4: top 20 altered gene clusters identified in the 232 deregulated genes that are significantly associated with OS. Gene set enrichment analysis was conducted using Metascape (http://metascape.org). Count represents the number of genes with membership in the given ontology term. “%” represents the percentage of total 232 genes associated with OS that are found in the given ontology term. Log10 (*p*) is the *p*-value in log base 10. Table S5: frequency by which each gene appeared in the Cox regression model among 100 resampling training sets. The signature genes are highlighted in yellow. Table S6: the function and role of 11 genes in the prognostic signature in normal and HGSOC cells. Table S7: the average Cox regression coefficient for each gene used to calculate the prognostic score.

## References

[1] Siegel R. L., Miller K. D., Jemal A. (2017). Cancer statistics, 2017. *CA: A Cancer Journal for Clinicians*.

[2] Reid B. M., Permuth J. B., Sellers T. A. (2017). Epidemiology of ovarian cancer: a review. *Cancer Biology & Medicine*.

[3] Kroeger P. T., Drapkin R. (2017). Pathogenesis and heterogeneity of ovarian cancer. *Current Opinion in Obstetrics and Gynecology*.

[4] Prat J. (2012). New insights into ovarian cancer pathology. *Annals of Oncology*.

[5] Vang R., Shih I.-M., Kurman R. J. (2009). Ovarian low-grade and high-grade serous carcinoma. *Advances in Anatomic Pathology*.

[6] Cancer Genome Atlas Research Network (2011). Integrated genomic analyses of ovarian carcinoma. *Nature*.

[7] Matulonis U. A., Sood A. K., Fallowfield L. (2016). Ovarian cancer. *Nature Reviews Disease Primers*.

[8] Konecny G. E., Wang C., Hamidi H. (2014). Prognostic and therapeutic relevance of molecular subtypes in high-grade serous ovarian cancer. *JNCI: Journal of the National Cancer Institute*.

[9] Paik S., Shak S., Tang G. (2004). A multigene assay to predict recurrence of tamoxifen-treated, node-negative breast cancer. *New England Journal of Medicine*.

[10] Kratz J. R., He J., Van Den Eeden S. K. (2012). A practical molecular assay to predict survival in resected non-squamous, non-small-cell lung cancer: development and international validation studies. *The Lancet*.

[11] Wang P., Wang Y., Hang B. (2016). A novel gene expression-based prognostic scoring system to predict survival in gastric cancer. *Oncotarget*.

[12] Chen E. G., Wang P., Lou H. (2018). A robust gene expression-based prognostic risk score predicts overall survival of lung adenocarcinoma patients. *Oncotarget*.

[13] Spentzos D., Levine D. A., Ramoni M. F. (2004). Gene expression signature with independent prognostic significance in epithelial ovarian cancer. *Journal of Clinical Oncology*.

[14] Bonome T., Levine D. A., Shih J. (2008). A gene signature predicting for survival in suboptimally debulked patients with ovarian cancer. *Cancer Research*.

[15] Liu L. W., Zhang Q., Guo W., Qian K., Wang Q. (2016). A five-gene expression signature predicts clinical outcome of ovarian serous cystadenocarcinoma. *BioMed Research International*.

[16] Tothill R. W., Tinker A. V., George J. (2008). Novel molecular subtypes of serous and endometrioid ovarian cancer linked to clinical outcome. *Clinical Cancer Research*.

[17] Győrffy B., Lanczky A., Szallasi Z. (2012). Implementing an online tool for genome-wide validation of survival-associated biomarkers in ovarian-cancer using microarray data from 1287 patients. *Endocrine-Related Cancer*.

[18] Sfakianos G. P., Iversen E. S., Whitaker R. (2013). Validation of ovarian cancer gene expression signatures for survival and subtype in formalin fixed paraffin embedded tissues. *Gynecologic Oncology*.

[19] Gyorffy B., Surowiak P., Budczies J., Lánczky A. (2013). Online survival analysis software to assess the prognostic value of biomarkers using transcriptomic data in non-small-cell lung cancer. *PLoS One*.

[20] Tripathi S., Pohl M. O., Zhou Y. (2015). Meta- and orthogonal integration of influenza “OMICs” data defines a role for UBR4 in virus budding. *Cell Host & Microbe*.

[21] Bindea G., Mlecnik B., Hackl H. (2009). ClueGO: a cytoscape plug-in to decipher functionally grouped gene ontology and pathway annotation networks. *Bioinformatics*.

[22] Goenawan I. H., Bryan K., Lynn D. J. (2016). DyNet: visualization and analysis of dynamic molecular interaction networks. *Bioinformatics*.

[23] Sparano J. A., Gray R. J., Makower D. F. (2018). Adjuvant chemotherapy guided by a 21-gene expression assay in breast cancer. *New England Journal of Medicine*.

[24] Cardoso F., van’t Veer L. J., Bogaerts J. (2016). 70-gene signature as an aid to treatment decisions in early-stage breast cancer. *New England Journal of Medicine*.

[25] Chudasama D., Bo V., Hall M. (2018). Identification of cancer biomarkers of prognostic value using specific gene regulatory networks (GRN): a novel role of RAD51AP1 for ovarian and lung cancers. *Carcinogenesis*.

[26] Tanaka S., Tsuda N., Kawano K. (2000). Expression of tumor-rejection antigens in gynecologic cancers. *Japanese Journal of Cancer Research*.

[27] He J., Liu Y., Zhang L., Zhang H. (2018). Integrin subunit beta 8 (ITGB8) upregulation is an independent predictor of unfavorable survival of high-grade serous ovarian carcinoma patients. *Medical Science Monitor*.

[28] Zhang Q., Burdette J. E., Wang J. P. (2014). Integrative network analysis of TCGA data for ovarian cancer. *BMC Systems Biology*.

[29] Nguyen L. N., Holdren M. S., Nguyen A. P. (2006). Sorting nexin 1 down-regulation promotes colon tumorigenesis. *Clinical Cancer Research*.

[30] Ju W., Yoo B. C., Kim I. J. (2009). Identification of genes with differential expression in chemoresistant epithelial ovarian cancer using high-density oligonucleotide microarrays. *Oncology Research Featuring Preclinical and Clinical Cancer Therapeutics*.

[31] Wang J., Chen C., Li H. F., Jiang X. L, Zhang L. (2016). Investigating key genes associated with ovarian cancer by integrating affinity propagation clustering and mutual information network analysis. *European Review for Medical and Pharmacological Sciences*.

[32] Liu L., Xiong Y., Xi W. (2017). Prognostic role of *N*-Acetylgalactosaminyltransferase 10 in metastatic renal cell carcinoma. *Oncotarget*.

[33] Zhan X.-Y., Zhang Y., Zhai E., Zhu Q.-Y., He Y. (2018). Sorting nexin-1 is a candidate tumor suppressor and potential prognostic marker in gastric cancer. *PeerJ*.

[34] Koseki J., Konno M., Asai A. (2018). Enzymes of the one-carbon folate metabolism as anticancer targets predicted by survival rate analysis. *Scientific Reports*.

[35] Lin H., Huang B., Wang H. (2018). MTHFD2 overexpression predicts poor prognosis in renal cell carcinoma and is associated with cell proliferation and vimentin-modulated migration and invasion. *Cellular Physiology and Biochemistry*.

[36] Noguchi K., Konno M., Koseki J. (2018). The mitochondrial one-carbon metabolic pathway is associated with patient survival in pancreatic cancer. *Oncology Letters*.

[37] Liao W. C., Liao C. K., Tsai Y. H. (2018). DSE promotes aggressive glioma cell phenotypes by enhancing HB-EGF/ErbB signaling. *PLoS One*.

[38] Ata R., Antonescu C. N. (2017). Integrins and cell metabolism: an intimate relationship impacting cancer. *International Journal of Molecular Sciences*.

[39] Lee J.-M., Nair J., Zimmer A. (2018). Prexasertib, a cell cycle checkpoint kinase 1 and 2 inhibitor, in BRCA wild-type recurrent high-grade serous ovarian cancer: a first-in-class proof-of-concept phase 2 study. *The Lancet Oncology*.

