# Supplementary material for: A Robust Gene Expression Prognostic Signature for Overall Survival in High-Grade Serous Ovarian Cancer
Source: J Oncol. 2019 Nov 7;2019:3614207. doi: 10.1155/2019/3614207 (PMC6925684; doi:10.1155/2019/3614207)
Supplement: Supplementary Materials — Table S1: summary of independent validation of the 11-gene signature in 9 datasets. Table S2: list of genes that are consistently deregulated in HGSOC across six datasets using criteria: adjusted p < 0.05 and fold change >1.5. Table S3: the impact of deregulated genes on overall survival (OS). Genes significantly associated with OS are highlighted in yellow. Table S4: top 20 altered gene clusters identified in the 232 deregulated genes that are significantly associated with OS. Gene set enrichment analysis was conducted using Metascape (http://metascape.org). Count represents the number of genes with membership in the given ontology term. “%” represents the percentage of total 232 genes associated with OS that are found in the given ontology term. Log10 (p) is the p-value in log base 10. Table S5: frequency by which each gene appeared in the Cox regression model among 100 resampling training sets. The signature genes are highlighted in yellow. Table S6: the function and role of 11 genes in the prognostic signature in normal and HGSOC cells. Table S7: the average Cox regression coefficient for each gene used to calculate the prognostic score. [file 3614207.f1.zip › 3614207.f1/Supplementary Table 1.docx]

**Table S1.** Summary of independent validation of the 11-gene signature with 9 datasets

| Name of  datasets | P  Value | HR | 95%CI for HR | |
| --- | --- | --- | --- | --- |
|  |  |  | Lower | Upper |
| GSE32063 | 0.000269 | 10.39 | 2.95 | 36.61 |
| GSE19829 GPL570 | 0.003775 | 5.81 | 1.77 | 19.11 |
| GSE30161 | 0.000993 | 3.4 | 1.64 | 7.05 |
| GSE3149 | 0.000279 | 3.12 | 1.69 | 5.76 |
| OV-AU-ICGC* | 7.57E-06 | 3.3 | 1.96 | 5.56 |
| GSE14764 | 0.002578 | 2.31 | 1.34 | 3.99 |
| GSE9891 | 0.000116 | 2.17 | 1.47 | 3.23 |
| GSE17260 | 0.03179 | 1.73 | 1.05 | 2.87 |
| GSE32062 | 0.01977 | 1.54 | 1.07 | 2.22 |

*Indicates dataset “OV-AU - ICGC Ovarian Cancer-Serous cystadenocarcinoma - June 2016”
