# Supplementary material for: A Robust Gene Expression Prognostic Signature for Overall Survival in High-Grade Serous Ovarian Cancer
Source: J Oncol. 2019 Nov 7;2019:3614207. doi: 10.1155/2019/3614207 (PMC6925684; doi:10.1155/2019/3614207)
Supplement: Supplementary Materials — Table S1: summary of independent validation of the 11-gene signature in 9 datasets. Table S2: list of genes that are consistently deregulated in HGSOC across six datasets using criteria: adjusted p < 0.05 and fold change >1.5. Table S3: the impact of deregulated genes on overall survival (OS). Genes significantly associated with OS are highlighted in yellow. Table S4: top 20 altered gene clusters identified in the 232 deregulated genes that are significantly associated with OS. Gene set enrichment analysis was conducted using Metascape (http://metascape.org). Count represents the number of genes with membership in the given ontology term. “%” represents the percentage of total 232 genes associated with OS that are found in the given ontology term. Log10 (p) is the p-value in log base 10. Table S5: frequency by which each gene appeared in the Cox regression model among 100 resampling training sets. The signature genes are highlighted in yellow. Table S6: the function and role of 11 genes in the prognostic signature in normal and HGSOC cells. Table S7: the average Cox regression coefficient for each gene used to calculate the prognostic score. [file 3614207.f1.zip › 3614207.f1/Supplementary Table 6.docx]

**Table S6**. The function and role of 11 genes in normal and cancer cells.

| **Gene &**  **Protein** | **NM** | **Main function** | **Role in ovarian cancer** | **Role in other types of cancer** |
| --- | --- | --- | --- | --- |
| *RAD51AP1*  (RAD51 associated protein 1) | NM_  006479 | Involved in homologous recombination and double strand DNA repair. | High expression  in ovarian cancer, which was associated  with poor OS. | Over-expressed  in lung cancer. |
| *CADPS2*  (Ca^2+^-dependent activator protein for secretion 2) | NM_  017954 | A calcium binding protein regulating the exocytosis of synaptic and dense-core vesicles in neurons and neuroendocrine cells. | Not reported. | Not reported. |
| *DSE* (dermatan sulfate epimerase) | NM-  013352 | Encodes a cancer cell antigen in the endoplasmic reticulum with functions in biosynthesis of dermatan sulfate. It has tumor epitopes for inducing HLA-A24-restricted and tumor- specific T-cells in cancer. | Elevated levels in cervical, ovarian and endometrial cancers. | (1) Frequently upregulated in brain tumors and other types of cancer such as squamous cell carcinoma and colorectal cancer.  (2) Elevated expression in glioma is associated with poor OS |
| *ITGB8* (integrin subunit beta 8) | NM_  002214 | A transmembrane receptor subunit. Mediate cell-cell and cell-extracellular matrix interactions. | Upregulated and predict poor survival in HGSOCs. | Upregulated in some other types of cancer, *e.g*., lung. |
| *PDE10A*  (phospho-  diesterase 10A) | NM_  001130690 | A cyclic nucleotide phosphodiesterase family member and plays a role in signal transduction by regulating the intracellular concentration of cyclic nucleotides. | Not reported. | may play a role in the predisposition and/or progression to prostate cancer. |
| *GALNT10*  (polypeptide N-acetylgalactosaminyltrans-  ferase 10) | NM_  003099 | The encoded enzyme catalyzes the first step in the  glycoprotein synthesis. | Highly predictive for the overall survival. | May play a prognostic role in renal cell carcinoma. |
| *SNX1* (sorting nexin 1) | NM_  003099 | Encodes a member of the sorting nexin family. Involved in intracellular trafficking and signaling. | Potential tumor suppressor, and down-regulated in chemo-  resistant ovarian cancers. | Potential prognostic biomarker in gastric and colorectal cancer. |
| *MTHFD2*  (methylenetetrahydrofolate dehydrogenase) | NM_  006638 | Encodes a mitochondrial enzyme involved in one-carbon folate metabolism. | Not reported. | (1) Associated with tumor cell proliferation and development.(2) Increased expression correlates with poor prognosis of many types of cancer, including breast, lung, liver, colorectal, renal, CNS tumors. |
| *C9orf16*  (chromosome 9 open reading frame 16) | NM_  024112 | A member of the UPF0184 family. | A key gene in the progress of ovarian cancer, according to one  network-based approach-based study. | Not reported. |
| *PYCR1* (pyrroline-5-carboxylate reductase 1) | NM_  006907 | Housekeeping enzyme that catalyzes the last step in proline biosynthesis. Involved in the cellular response to oxidative stress. | Not reported. | Promote cancer proliferation and progression. |
| *ARL4* (ADP ribosylation factor-like 4) | NM_  005737 | Encodes a member of the ADP-ribosylation factor family of GTP-binding proteins. | Not reported. | Not reported. |
